# Dissociating frontal regions that co-lateralize with different ventral occipitotemporal regions during word processing^☆^

**DOI:** 10.1016/j.bandl.2013.04.003

**Published:** 2013-08

**Authors:** Mohamed L. Seghier, Cathy J. Price

**Affiliations:** Wellcome Trust Centre for Neuroimaging, Institute of Neurology, UCL, London, UK

**Keywords:** Functional MRI, Language, Word processing, Semantic matching, Laterality index, Left lateralization, Inter-subject variability, Language subsystems

## Abstract

The ventral occipitotemporal sulcus (vOT) sustains strong interactions with the inferior frontal cortex during word processing. Consequently, activation in both regions co-lateralize towards the same hemisphere in healthy subjects. Because the determinants of lateralisation differ across posterior, middle and anterior vOT subregions, we investigated whether lateralisation in different inferior frontal regions would co-vary with lateralisation in the three different vOT subregions. A whole brain analysis found that, during semantic decisions on written words, laterality covaried in (1) posterior vOT and the precentral gyrus; (2) middle vOT and the pars opercularis, pars triangularis, and supramarginal gyrus; and (3) anterior vOT and the pars orbitalis, middle frontal gyrus and thalamus. These findings increase the spatial resolution of our understanding of how vOT interacts with other brain areas during semantic categorisation on words.

## Highlights

• Co-variation in lateralization during word reading dissociated three subsystems.
• Posterior ventral occipito-temporal cortex (vOT) with precentral gyrus.
• Middle vOT with pars opercularis, pars triangularis and supramarginal gyrus.
• Anterior vOT with pars orbitalis, middle frontal gyrus and thalamus.

## 1 Introduction

The lateralisation of cognitive functions in the human brain illustrates how processing is efficiently distributed across the left and right hemispheres (Hugdahl, 2000). This is because processing efficiency is thought to be greater when cerebral regions supporting a given function are in the same hemisphere (Ringo, Doty, Demeter, & Simard, 1994). It follows that cerebral regions that have activation similarly lateralized to the same hemisphere may be part of the same functional subsystem.

In the reading domain, it has been shown that activation is lateralized to the dominant (left) hemisphere during word processing even in the most posterior parts of the reading system, such as the ventral occipito-temporal sulcus (vOT) (Dehaene & Cohen, 2007; Dien, 2009). One hypothesis posits that left-lateralized activity in vOT results from its strong interactions with the left-lateralized frontal language regions (Cai, Lavidor, Brysbaert, Paulignan, & Nazir, 2008). For instance, previous fMRI studies have shown that, during word processing, vOT consistently lateralises to the same hemisphere as the inferior frontal gyrus (e.g. (Cai, Paulignan, Brysbaert, Ibarrola, & Nazir, 2010; Van der Haegen, Cai, & Brysbaert, 2012)). Here we aim to examine this relationship, with high spatial definition, in healthy skilled readers performing semantic decisions on written words. We also tested whether lateralization in other brain regions outside the inferior frontal gyrus covaried with lateralization in vOT.

Our approach differs from previous studies at several levels. First, our analyses are conducted at high spatial definition. Previous reports have investigated the co-lateralization between vOT and the inferior frontal gyrus with the assumption that both regions are spatially homogenous. This is typically done by computing laterality over large volumes of interest in vOT and the inferior frontal gyrus. However, many studies have demonstrated a strong functional heterogeneity in both vOT (e.g. (Moore & Price, 1999; Price & Mechelli, 2005; Seghier, Lee, Schofield, Ellis, & Price, 2008; Seghier & Price, 2011; Szwed et al., 2011; Vinckier et al., 2007)) and the inferior frontal gyrus (e.g. (Amunts et al., 2010; Anwander, Tittgemeyer, von Cramon, Friederici, & Knösche, 2007; Buckner, Raichle, & Petersen, 1995; Friederici, Opitz, & von Cramon, 2000; Juch, Zimine, Seghier, Lazeyras, & Fasel, 2005; Poldrack et al., 1999)). Furthermore, it has been shown that functional interactions between vOT and the inferior frontal gyrus can vary with the subregion tested, with posterior to anterior vOT subregions interacting with different frontal regions; see examples in (Mechelli et al., 2005; Seghier et al., 2008; van der Mark et al., 2011). We therefore predicted that laterality in different vOT subregions would correlate with laterality in different parts of the inferior frontal gyrus. This was investigated by correlating laterality at every brain voxel (Liegeois et al., 2002) with specific regions of interest in posterior, middle and anterior parts of vOT.

Our whole brain approach differs from the previous hypothesis-driven co-laterality approaches that were constrained to pre-defined vOT and inferior frontal regions (e.g. (Cai et al., 2010; Pinel & Dehaene, 2010; Van der Haegen et al., 2012)). These constrained analyses ignore the relationship between vOT laterality and brain regions that are outside the inferior frontal gyrus but still play an important role in word processing (see recent review in Price, 2012). Thus, we explicitly assessed correlations between laterality in vOT and laterality in all brain regions, with the expectation that word processing in other regions, outside the inferior frontal gyrus, may result in lateralized responses that were strongly correlated to laterality in vOT.

Having established how laterality in different vOT subregions varies with lateralization in other brain regions, we conducted additional post hoc checks to determine (a) whether left lateralization in each area was driven by positive left hemisphere activation, negative right hemisphere activation or both; and (b) whether laterality in each identified region co-lateralized towards the same hemisphere as the vOT subregion being tested. Indeed, as discussed by Van der Haegenand colleagues (2012), a significant correlation in laterality does not necessarily mean that both regions are lateralized in the same hemisphere. For instance, although vOT and frontal regions were left-lateralized during sentence reading, there was no significant correlation between their laterality indices (Pinel & Dehaene, 2010). Thus, we also generated here co-lateralization maps at the voxel level to complement our across-subject correlation analyses.

The above analyses were conducted in data acquired when skilled readers were asked to make semantic decisions on familiar written object names. We selected this task because semantic decisions yield robust and consistent lateralization patterns both at the group as well as the subject level (e.g. (Lehéricy et al., 2000; Seghier et al., 2004)). For instance, we previously showed that laterality in different parts of vOT was greater during semantic decisions than reading aloud even when the word stimuli were held constant (Seghier & Price, 2011). Indeed, because word processing in vOT is likely to be task-dependent (e.g. (Guo & Burgund, 2010; Large, Aldcroft, & Vilis, 2007)), laterality is likely to vary in studies that used lexical decision (Cai et al., 2010; Van der Haegen et al., 2012) or sentence reading (Pinel & Dehaene, 2010). Thus, our laterality analyses were conducted on data collected during a semantic matching task that had already been shown to generate robust left-lateralized patterns in vOT and other word processing areas.

## 2 Materials and methods

### 2.1 Subjects

Here we used a subset of the data used in our previous work (Josse, Seghier, Kherif, & Price, 2008; Seghier & Price, 2011) that investigated the condition dependent determinants of lateralization in the same three vOT subregions we investigate here. These data were collected in 82 healthy subjects (43 females, 39 males, 30.3 ± 15 years old, 44 right-handed, 38 left-handed or ambidextrous). Subjects were native English speakers, had normal or corrected-to-normal vision, and had no history of neurological or psychiatric disorders. The study was approved by the National Hospital for Neurology and Institute of Neurology Joint Ethic’s Committee.

### 2.2 Stimuli and tasks

There were 4 different stimuli: written names of objects, pictures of objects, unfamiliar Greek strings, and unfamiliar nonobjects. All stimuli were presented in triads with one item (picture or letter string) above and two items below in the same format as the item above. In 2 separate scanning runs or sessions, the participants made semantic and perceptual decisions, interleaved with blocks of fixation. During semantic and perceptual decisions, the item above acted as a target that was semantically or physically related to one of the items below, and subjects cued a finger press to indicate their responses. Prior to each stimulus block, a brief instruction was presented on the screen for 3.6 s to indicate what sort of response would be necessary. The order of conditions was counterbalanced within and across session. Each session consisted of 24 blocks of stimuli of the same type/condition with an additional 12 blocks of fixation that were presented every two stimulus blocks. Each stimulus block lasted 18 s and consisted of 4 trials during which 3 stimuli were simultaneously presented on the screen for 4.32 s, followed by 180 ms of fixation. Every two stimulus blocks, fixation continued for 14.4 s. Stimulus presentation in the scanner was via a video projector, a front-projection screen and a system of mirrors fastened to the MRI head coil.

**Fig. 1:**
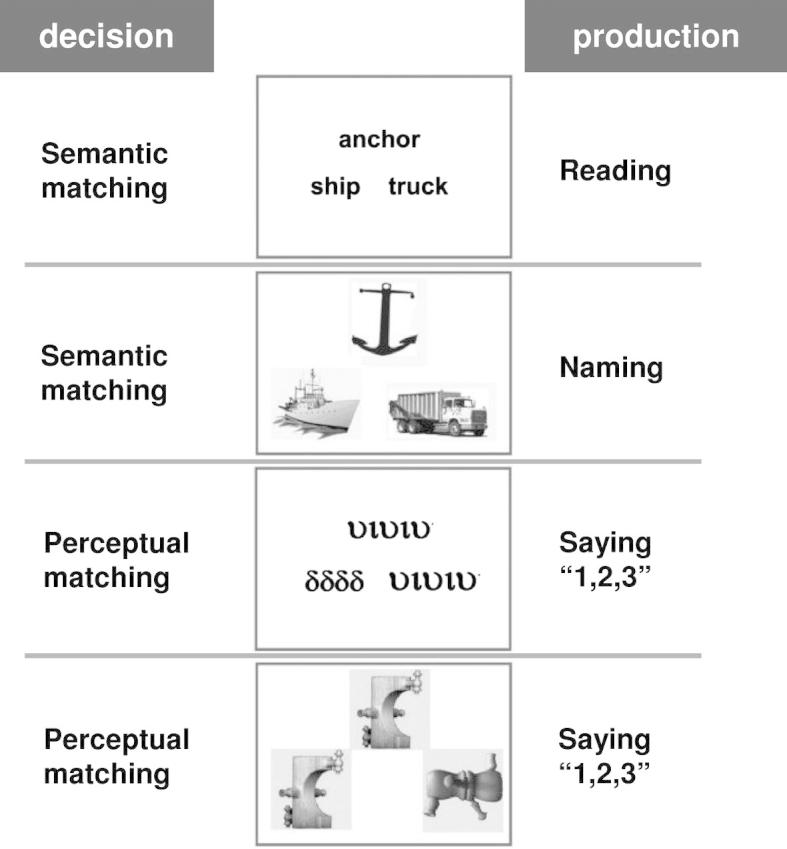
Illustrates the different experimental conditions. The two decision sessions included semantic matching on words and pictures, and perceptual matching on unfamiliar Greek letters and nonobjects. The two production sessions included reading aloud, object naming and saying “1, 2, 3” to unfamiliar Greek letters and nonobjects. All conditions were interleaved with blocks of fixation.

In addition to the two experimental sessions that each involved the semantic and perceptual decisions described above, our study also included two sessions of speech production that are not reported here. The speech production conditions involved naming pictures of familiar objects, reading aloud written names of familiar objects and saying “1, 2, 3” to meaningless and unfamiliar Greek strings and nonobjects. Fig. 1 illustrates the different tasks and stimuli. The current study only focuses on semantic decisions on written words (see below). Additional details about the paradigm and stimuli can be found in our previous work (c.f. (Josse, Kherif, Flandin, Seghier, & Price, 2009; Seghier, Fagan, & Price, 2010; Seghier & Price, 2011)).

### 2.3 MRI acquisition

Experiments were performed on a 1.5T Siemens system (Siemens Medical Systems, Erlangen, Germany). Functional imaging consisted of an EPI GRE sequence (TR/TE/flip angle = 3600 ms/50 ms/90°, FOV = 192 mm, matrix = 64 × 64, 40 axial slices, 2 mm thick with 1 mm gap). Anatomical T1-weighted images were acquired using a three-dimensional modified driven equilibrium Fourier transform sequence (TR/TE/TI = 12.24 ms/3.56 ms/530 ms, matrix = 256 × 224, 176 sagittal slices with a final resolution of 1 mm^3^).

### 2.4 fMRI data preprocessing

Data processing and statistical analyses were performed with the Statistical Parametric Mapping SPM5 software package (Wellcome Trust Centre for Neuroimaging, London, UK). All functional volumes were spatially realigned, un-warped, and normalised to the MNI space using the unified normalisation-segmentation procedure, with resulting voxels size of 2 × 2 × 2 mm^3^. During the normalisation-segmentation step, symmetrical priors were used. The resulting normalisation-segmentation parameters were then applied to the subject’s functional images thereby rendering them symmetrical, which allows left and right hemisphere activation to be directly compared (Josse et al., 2009; Seghier, Kherif, Josse, & Price, 2011). The normalised (symmetrical) functional images were then spatially smoothed with a 6 mm full width half maximum isotropic Gaussian kernel.

### 2.5 First level analyses

For each individual subject, we carried out a fixed-effect analysis on all pre-processed functional volumes of that subject, using the general linear model at each voxel. Time-series from each voxel were high-pass filtered (1/128 Hz cut-off) to remove low frequency noise and signal drift. Each stimulus onset was modelled as an event in condition-specific ‘stick-functions’ with a duration of 4.32 s per trial and a stimulus onset interval of 4.5 s. Correct responses for each condition, instructions, and errors were modelled separately in the design matrix. The resulting stimulus functions were convolved with a canonical hemodynamic response function which provided regressors for the linear model. Therefore, for each session, the design matrix included separate regressors that coded instructions and both correct and incorrect trials for each one of the four conditions (Fig. 1). As in standard SPM analyses, the design matrix also included four (constant) regressors to model the average signal in each session. Summary or contrast images for the regressor coding correct trials during semantic decisions on words relative to fixation were generated in all subjects.

### 2.6 Voxel based laterality maps

Rather than using global or regional lateralization indices, we created images of the lateralization score at each voxel across the entire brain. This was achieved by computing the relative difference in activity level between each voxel in the left hemisphere and its homologue in the right hemisphere. Such voxel-based laterality maps thus coded hemispheric differences for each task, at each voxel, for each subject (Baciu, Juphard, Cousin, & Bas, 2005; Josse et al., 2009; Liegeois et al., 2002; Nakamura et al., 2005; Seghier & Price, 2011; Seghier et al., 2011; Xue et al., 2005). Put another way, laterality maps code the interaction between task (activation versus control) and hemisphere (left versus right) at each voxel (Liegeois et al., 2002; Seghier & Price, 2011). It is worth noting that, unlike laterality indices that can be generated at the subject level (Seghier, 2008), voxel-based laterality maps are typically assessed over subjects at the group level (Liegeois et al., 2002).

### 2.7 Whole-brain covariance analysis

Laterality scores in vOT were included as covariates of interest in multiple regression analyses that included either whole brain laterality maps or the original whole brain contrast images. The vOT laterality values were extracted using the VOI tool of SPM at three predefined vOT subregions. These three subdivisions used here as seed regions were identified on the basis of our previous work on the functional determinants of laterality in different vOT subregions (Seghier & Price, 2011) as follows: posterior vOT (pvOT) at MNI-coordinates [*x* = −42, *y* = −70, *z* = −10], middle vOT (mvOT) at [*x* = −44, *y* = −54, *z* = −16], and anterior vOT (avOT) at [*x* = −44, *y* = −44, *z* = −16]. For each vOT seed region, laterality values (principal eigenvariates) were extracted within a 4 mm-diameter sphere centred at the MNI coordinate of each vOT subregion. Our covariance analysis searched across the whole brain for where laterality varied similarly across subjects with the laterality in the seed region (e.g. pvOT, mvOT or avOT), see for a similar rationale (Seghier et al., 2008). To examine the direction of covariance in laterality in more detail, we also correlated the degree to which vOT activation was left lateralized with activation (not lateralization) in each hemisphere separately. Significant results are reported at *p* < 0.001 with correction for multiple comparisons (p-FWE < 0.05) made on the basis of height or extent.

### 2.8 Voxel-based co-lateralization maps

As mentioned above, significant correlations in laterality does not always mean co-lateralization to the same hemisphere (for a discussion see (Van der Haegen et al., 2012)). We thus investigated whether regions that strongly correlated with vOT laterality in the covariance analysis above also co-lateralized to the same hemisphere as vOT. Practically, the co-lateralization analysis typically counts the number of subjects who have both seed and target regions lateralized to the same hemisphere. Previous studies have used regional laterality indices to assess co-lateralization (e.g. (Cai et al., 2010; Van der Haegen et al., 2012)). Such laterality indices typically measure the relative difference between left and right hemisphere activation within a relatively large volume of interest in each individual subject (see review in Seghier, 2008). Here we generated such laterality indices when the size of the volumes of interest is equal to one voxel only. Each voxel can either be defined as a binary quantity (e.g. from a threshoded *t*-map as commonly used in regional laterality indices) or as a continuous value that codes the amplitude of the activation (i.e. parameter estimates) for a given task. Here we used the later because this was already coded in our voxel-based laterality maps as explained above. In this context, a laterality index at a given voxel is set to +1 when left activation is higher than right activation and to −1 for the opposite effect. Thus, for each subject, the final image was a signed lateralization map that displayed either +1 or −1 at each voxel of the whole hemisphere. Note that such signed (binary) lateralization maps are equivalent to the categorical laterality indices that are typically generated after thresholding the regional laterality indices (e.g. typically thersholded at 0.2 (Springer et al., 1999) or even at 0.5 as in (Cai et al., 2010; Van der Haegen et al., 2012)).

We then computed, at each voxel of the whole brain, the number of subjects that have the same sign as a given vOT subregion. This number can be divided by the total of number of subjects to measure the proportion of subjects with identical co-lateralization in each voxel with a given vOT subregion. In other words, the generated co-lateralization map codes, at each voxel, the proportion of subjects that lateralized to the same hemisphere as a given vOT subregion. It is worth noting that our procedure is more conservative than previous co-lateralization analyses based on regional laterality indices because the volume of interest was reduced here to one voxel only. In addition, the inclusion of left-handed subjects may also yield lower co-lateralization scores, as shown previously when comparing right-handers to left-handers (Cai et al., 2010; Van der Haegen et al., 2012). Last but not least, in our procedure, co-lateralisation is assessed at each voxel and across the whole brain, which allows the search for other co-lateralized effects outside the inferior frontal gyrus.

## 3 Results

**Fig. 2:**
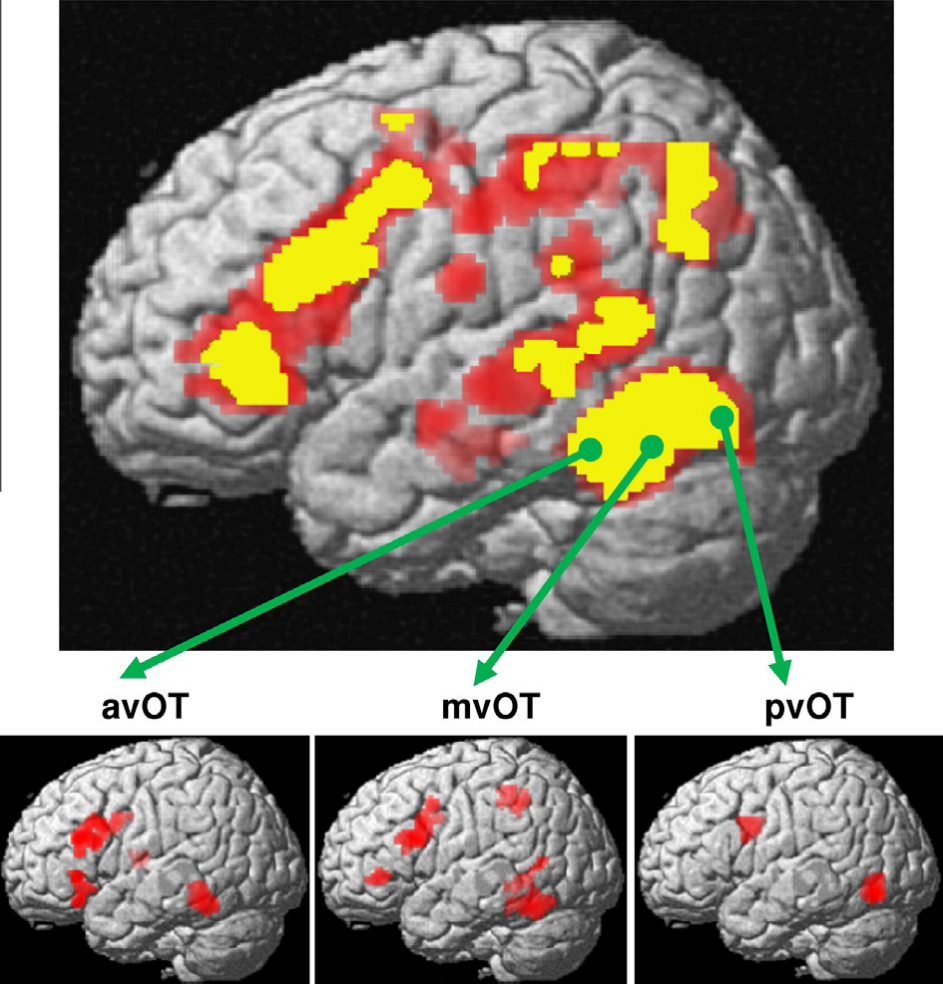
(Top) 3D rendering of left-lateralized voxels for semantic matching on words versus fixation. This figure illustrates the group main effect over the voxel-based laterality maps at *p* < 0.05 FWE-corrected over the whole brain (in yellow) and at a lower threshold *p* < 0.001 uncorrected (in red). (Bottom) second-level covariance analysis on voxel based laterality maps. This figure illustrates all voxels (in red) where laterality significantly correlated, across our 82 subjects, with laterality in any of the three seed regions pvOT, mvOT and avOT, during semantic matching on words. Significant voxels are shown at *p* < 0.05 FWE-corrected over the whole brain. pvOT = posterior vOT at MNI-coordinates [*x* = −42, *y* = −70, *z* = −10]; mvOT = middle vOT at [*x* = −44, *y* = −54, *z* = −16]; avOT = anterior vOT at [*x* = −44, *y* = −44, *z* = −16].

Our whole brain covariance analysis on voxel-based laterality maps revealed strong correlations in laterality between vOT and the inferior frontal gyrus (Fig. 2), in line with previous work (Cai et al., 2010; Van der Haegen et al., 2012). As predicted, vOT subregions correlated differentially with distinct inferior frontal subregions, see full list of coordinates in Table 1. More specifically, at *p* < 0.05 FWE-corrected, lateralization in pars opercularis and pars triangularis was strongly related to lateralization in mvOT, whereas lateralization in pars orbitalis was strongly related to lateralization in avOT with this effect being stronger with avOT than mvOT (*Z* = 3.9) or pvOT (*Z* = 2.8). The significant laterality correlations were a consequence of both greater left hemisphere activation (*Z* = 4.6 in left pars opercularis, *Z* = 2.7 in left triangularis, and *Z* = 2.9 in left orbitalis) and reduced activation in their homologue right regions (*Z* = 2.7 in right pars opercularis, *Z* = 2.3 in right triangularis, and *Z* = 2.6 in right orbitalis).

Interestingly, other frontal regions also showed significant correlations with laterality in vOT. This included (i) laterality in the precentral gyrus correlating with laterality in pvOT, with this effect being primarily driven by reduced activation in the right precentral gyrus (*Z* = 4.3) rather than greater activation in left precentral gyrus (*Z* = 1.9), and (ii) laterality in the middle frontal gyrus correlating with laterality in avOT, with this effect being driven by both increased left (*Z* = 4.4) and reduced right (*Z* = 4.0) hemisphere activation.

Outside the frontal lobe (at p < 0.05 FWE-corrected, Table 1), (i) laterality in dorsal supramarginal gyrus correlated with laterality in mvOT, as a result of greater left (*Z* = 5.2) than right (*Z* = 1.8) hemisphere activation, and (ii) laterality in dorsomedial thalamus correlated strongly with laterality in avOT, with this effect being driven by both increased left (*Z* = 2.2) and reduced right (*Z* = 2.7) hemisphere activation. Finally, our co-lateralization analysis at the voxel level showed that, across subjects, all the significant clusters of Table 1 were co-lateralised towards the same hemisphere as the vOT subregions. Across regions, this was consistent for 67% to 85% of our skilled readers (Table 2).

## 4 Discussion

Co-lateralization of reading activation in vOT and the inferior frontal gyrus has previously been reported in both left- and right-handers (Cai et al., 2010; Van der Haegen et al., 2012). Our findings confirm this relationship and were able to provide higher spatial definition than previous studies because we used a voxel based analysis rather than large regions of interest. This allowed us to show that laterality in different vOT subdivisions covaries with laterality in different frontal subdivisions. In addition, by using a whole-brain unconstrained search, we were able to show that laterality in vOT correlated with laterality in other regions outside the frontal lobe. The sets of areas associated with different vOT regions segregate the semantic word processing network into multiple subsystems, on the basis of their differential correlation with region-dependent laterality in vOT. Below, we discuss the functions of the segregated subsystems and the implications that our results have for future studies.

The dissociation of different functional responses within both vOT and the inferior frontal cortex fits with many previous reports (e.g. (Amunts et al., 2010; Anwander et al., 2007; Friederici et al., 2000; Juch et al., 2005; Moore & Price, 1999; Seghier & Price, 2011; Seghier et al., 2008; Szwed et al., 2011; van der Mark et al., 2011; Vinckier et al., 2007)). For example, using the same data, we previously found (Seghier & Price, 2011) that pvOT lateralization is influenced by the spatial frequency of the visual inputs, avOT lateralization is influenced by the semantic demands of the task and mvOT lateralization is influenced by a combination of visual expertise and semantics. These previous reports focused on task and stimulus manipulations whereas the current analysis looked at co-variations among different regions during a single task – semantic categorisations on written words.

The interaction of different vOT subdivisions that we found with different inferior frontal subdivisions is remarkably consistent with the dynamic causal modelling study reported by Mechelli et al. (2005). In their study, Mechelli et al. showed that effective connectivity between posterior vOT and dorsal frontal regions was stronger for non-semantic reading (pseudowords more than words with irregular spellings); while effective connectivity between anterior vOT and ventral frontal cortex was stronger for semantic reading (irregularly spelled words more than pseudowords). Here our correlations in laterality (Fig. 2 and Table 1) showed a similar pattern: laterality in pvOT strongly correlated with laterality in the precentral gyrus, laterality in avOT strongly correlated with laterality in pars orbitalis and laterality in mvOT strongly correlated with laterality in pars opercularis. In addition, we found that the avOT and pars orbitalis network also involved the dorsomedial thalamus and the middle frontal gyrus, while the mvOT and pars opercularis/pars triangularis network also involved the dorsal supramarginal gyrus. The combination of regions linked to each vOT subdivision gives clues to the function of each network but we acknowledge, *a priori*, that the function of a region may vary depending on the network that it activates in (Poldrack, 2006; Price & Friston, 2005), and each subsystem may be engaged in more than one function (see Kim, Karunanayaka, Privitera, Holland, & Szaflarski, 2011).

With respect to the mvOT network, we note that pars opercularis and pars triangularis are activated during both phonological and semantic processing (e.g. (Devlin, Matthews, & Rushworth, 2003)) while activation in the dorsal supramarginal gyri is typically associated with phonological more than semantic processing (e.g. (Church, Balota, Petersen, & Schlaggar, 2011; Hartwigsen et al., 2010; Sliwinska, Khadilkar, Campbell-Ratcliffe, Quevenco, & Devlin, 2012; Stoeckel, Gough, Watkins, & Devlin, 2009). Activation of these frontal and parietal regions during semantic decisions on written words may therefore be related to phonological processing that occurs implicitly during semantic decisions on written words and is not subtracted out when the baseline is fixation or perceptual matching. Other areas may mediate the interactions between mvOT, pars opercularis, pars triangularis and the dorsal supramarginal gyrus; for instance, using graph theory analysis to segregate different subsystems, Vandenberghe et al. (in press) have recently shown that a hub at the posterior middle temporal gyrus connects a hub at mvOT (*y* = −57 mm) to higher level (e.g. frontal) language regions (Vandenberghe et al., in press). In our study, the group main effect over the voxel-based laterality maps for semantic matching on words versus fixation was strongly left lateralized at [−58 −52 12] and [−56 −38 −4] (Fig. 2) but laterality in these regions did not co-vary with mvOT or any other vOT sub-region (Fig. 2).

With respect to the avOT network, all components are strongly associated with semantic rather than phonological processing. Specifically, (i) activation in the pars orbitalis is typically higher for semantic than phonological decisions (e.g. (Friederici et al., 2000; Mummery, Shallice, & Price, 1999; Poldrack et al., 1999)); (ii) recent lesion studies have associated focal damage to the medial thalamus with selective difficulties in semantic retrieval (Pergola et al., 2013), consistent with previous reports that thalamic damage impairs semantic processing (Cox & Heilman, 2011; Crosson, 1999; De Witte, Wilssens, Engelborghs, De Deyn, & Mariën, 2006); (iii) diffusion tractography in humans has shown anatomical interconnections between the mediodorsal nucleus of the thalamus and lateral orbitofrontal cortex (Draganski et al., 2008; Klein et al., 2010) consistent with these regions being part of the same subsystem and (iv) the semantic function of avOT during visual and auditory word processing is well recognised (for review see Price, 2012) and its association with pars orbitalis is consistent with the increased effective connectivity between avOT and ventral inferior frontal cortex during semantic reading tasks (Mechelli et al., 2005).

Interestingly, neither the current study or that reported by Mechelli et al. (2005) associated the middle temporal cortex with the avOT-ventral inferior frontal network, even though the middle temporal cortex is (i) consistently linked to semantic processing (for reviews see (Binder & Desai, 2011; Price, 2012)), (ii) strongly left lateralised across our participants (see Fig. 2), and (iii) co-lateralized to the same hemisphere as avOT in 83% of our subjects (Table 2). Indeed, we found that laterality in avOT was more correlated with laterality in the pars orbitalis than laterality in the two middle temporal clusters shown in Fig. 2: at [−56 −38 −4] (*Z* = 2.4, *p* = 0.008) and with a trend at [−58 −52 12] (*Z* = 1.5, *p* = 0.07). This suggests that these middle temporal regions are parts of functional subsystems that are not closely linked to our vOT regions of interest during semantic matching on words. For instance, Cornelissen et al. (2009) showed that MEG responses for words at a similar middle temporal cluster ([−50 −38 −2]) appeared at later time windows than the type of early responses occurred at vOT and inferior frontal gyrus. Thus, we can speculate that these middle temporal responses may occur too late to exert a significant impact on laterality in vOT. Other areas where activation was strongly and consistently left lateralised during semantic decisions but where the degree of lateralization did not correlate with that in one of the vOT regions are illustrated in Fig. 2 (e.g. the angular gyrus). Activation in these regions is therefore likely to be part of functional subsystems that have little influence on the lateralisation of the vOT regions we used to seed our covariance analyses.

With respect to the pvOT network, we found little evidence that this region was part of a language subsystem. The covariance analysis only linked laterality in pvOT to laterality in the precentral gyrus, with this effect being driven by reduced right precentral activation rather than increased left precentral activation. The absence of any covariance between laterality in pvOT and other left-lateralized language areas suggests that pvOT may not be influenced by linguistic factors. This is consistent with previous studies showing (i) the association of language dominance with anatomical asymmetries in middle/anterior vOT (around *y* = −50 mm) but not in posterior vOT (Greve et al., 2013); and (ii) laterality in pvOT being related to physical features (e.g. spatial frequency, see (Horie et al., 2012; Woodhead, Wise, Sereno, & Leech, 2011)) of the visual inputs rather than linguistic factors (Seghier & Price, 2011). Although pvOT laterality was not related to laterality in language processing areas, it is interesting to note how left-lateralized word activation emerges so early in the visual word processing stream. This has been reported previously with strong left-lateralized patterns identified at very posterior locations in the ventral, but not dorsal, visual system, even at coordinates *y* = −70 mm (Seghier & Price, 2011), *y* = −76 mm (Vigneau, Jobard, Mazoyer, & Tzourio-Mazoyer, 2005) and *y* = −80 mm (Szwed et al., 2011).

Above, we have described the likely functions of the subsystems that were dissociated on the basis of covariance in the strength of laterality. Other studies have investigated the subsystems of regions that contribute to semantic processing (Kim et al., 2011; Seghier & Price, 2009; Wu et al., 2009) but, here, our focus was on the subsystems that include avOT, mvOT or pvOT. Co-variation in laterality suggests that the regions involved are interacting with one another but it does not tell us how these interactions are implemented. Cai et al. (2008) and (2010) proposed that left-lateralized vOT activity is the consequence of interactive (top-down) processing from anterior (frontal) language areas (for a detailed discussion see also Price, 2012; Price & Devlin, 2011). This claim is supported by previous MEG studies that have shown that left inferior frontal activation occurs before, or in the same time frame, as left vOT activation during the first 200 ms of word processing (see for instance (Cornelissen et al., 2009; Pammer et al., 2004; Wheat, Cornelissen, Frost, & Hansen, 2010)). The hypothesis that vOT is influenced by top-down signals from the frontal cortex is also consistent with previous work that reported task dependent interactions between vOT and other left-lateralized regions (Levy et al., 2009; Mechelli et al., 2005; Richardson, Seghier, Leff, Thomas, & Price, 2011; Yvert, Perrone-Bertolotti, Baciu, & David, 2012) and fits with the interactive account of vOT function (Price & Devlin, 2011). Nevertheless, determining how different regions interact with one another needs to be tested with techniques, such as dynamic causal modelling , that indicate the directionality of information flow between regions.

The degree to which language activation is lateralized has been related to lateralisation in anatomical connections (e.g. (Powell et al., 2006). Therefore, the integration of anatomical and functional connectivity analyses may be useful for exploring whether distinct anatomical connections support the functional subsystems we have identified here. For example, several anatomical studies have described how vOT is richly connected with higher order language regions (Ben-Shachar, Dougherty, & Wandell, 2007; Vandermosten, Boets, Wouters, & Ghesquière, 2012; Yeatman, Rauschecker, & Wandell, 2012). These anatomical connections may explain intrinsic correlations in resting state-network analyses that have been observed between vOT and left frontal activation (e.g. see Fig. 1 of (Smith et al., 2009) and Fig. 2 of (Zhao et al., 2011)) and between mvOT and the dorsal supramarginal gyrus (Vogel, Miezin, Petersen, & Schlaggar, 2012). Our results raise new questions, for example, is avOT functionally or anatomically connected to the dorsomedial thalamus that activates with it?

In the current study, we used laterality of semantic activation to dissociate functional subsystems involving different vOT regions. When evaluating our results, we took into account that laterality is not an absolute attribute but reflects the relative difference between the two hemispheres. Any left-lateralized pattern can therefore be explained by either increased left hemisphere activation, decreased right hemisphere activation or both (Seghier et al., 2011); see for instance the contribution of both left and right vOT activation in defining laterality at vOT (Seghier & Price, 2011; Vigneau et al., 2005; Xue & Poldrack, 2007). Here, by correlating laterality in vOT with whole-brain left and right activation during semantic matching, we found that the majority of the significant correlations were a consequence not only of increased left hemisphere activation but also reduced right hemisphere activation. The only exception to this pattern was in the supramarginal gyrus where the correlation with laterality in mvOT was better explained by increased left hemisphere activation rather than reduced right hemisphere activation. This may reflect the important contribution that the right supramarginal gyrus plays in phonological processing (e.g. (Hartwigsen et al., 2010)). A systematic characterisation of laterality of this sort should eventually shed light on how functional specialisation emerges (Gazzaniga, 2000; Ringo et al., 1994) and why efficient processing is optimally enabled by lateralized networks (e.g. see simulations in (Shkuro & Reggia, 2003)). Both issues are also key to understanding the role of vOT in word processing (Dehaene & Cohen, 2007).

In summary, we have shown that laterality in different vOT subregions covaries with that in different left-lateralized language regions. These findings emphasise the fine-grained association between vOT and inferior frontal regions and dissociate different functional networks within the set of regions activated by semantic decisions on written words. To provide a mechanistic explanation for our findings, future anatomical and functional connectivity studies are needed to investigate regional interactions within and between the identified subsystems in each hemisphere. The impact of task on laterality for word stimuli also warrants further investigations. It would also be interesting to establish whether the functional associations obtained here with skilled English readers, generalise to other scripts (Hellige & Adamson, 2006; Xue et al., 2005) and populations. Last but not least, language laterality may change dynamically over the course of single word recognition (Khateb et al., 1999; Ortigue, Thut, Landis, & Michel, 2005) and thus the use of high temporal resolution techniques may shed some light on the exact time-course of interactivity between vOT subregions and the rest of the language/reading system (Cornelissen et al., 2009; Mainy et al., 2008; Pammer et al., 2004; Wheat et al., 2010).

## Figures and Tables

**Table 1:** Covariance analysis on voxel based laterality maps: list of coordinates (and *z* scores) of all regions that covaried with the different vOT subdivisions during semantic matching on words. At each coordinate, the *z*-scores of the differences in correlations between seed regions (here limited to the most significant effects) are reported. IFG = inferior frontal gyrus.

Region	Coordinates	vOT subregion		
		mvOT	avOT	pvOT
IFG: pars opercularis	−56 16 22	**5.1**	3.8	n.s.
	−40 14 20	**4.9**	3.7	n.s.
IFG: pars triangulartis	−52 34 0	**4.3**	3.1	n.s.
IFG: pars orbitalis	−42 26 −14	n.s.	**4.2**⁎⁎	n.s.
Middle frontal gyrus	−44 26 12	3.2	**4.3**	n.s.
	−50 28 22	3.2	**4.3**	3.8
Precentral gyrus	−38 4 30	3.8	3.9	**5.0**
Dorsomedial thalamus	−2 −8 10	n.s.	**5.0**	n.s.
Dorsal supramarginal gyrus	−34 −44 40	**4.7**	n.s.	n.s.
	−40 −42 50	**4.1**	3.2	n.s.

Bold = significant at *p* < 0.05 FWE-corrected over the whole brain; n.s. = not significant at *p* < 0.001 uncorrected.

⁎⁎ Significant region-by-laterality interaction at *p* < 0.001 uncorrected: avOT > mvOT (*Z* = 3.9).

**Table 2:** Co-lateralisation (CL) at each cluster identified in the covariance analysis (within the regions listed in Table 1). CL represents the proportion of subjects (out of 82 subjects) that lateralized to the same hemisphere as the seed subregion in vOT.

Region	Coordinates	CL	Other effects: maximum co-lateralization
*pvOT*			
Precentral gyrus	−38 4 30	0.73	Pars triangularis [−50 36 −4]; CL = 0.79

*mvOT*			
IFG: pars opercularis	−56 16 22	0.80	
	−40 14 20	0.80	
IFG: pars triangulartis	−52 34 0	0.85	Global maximum at the pars triangularis
Dorsal supramarginal gyrus	−34 −44 40	0.60	
	−40 −42 50	0.73	

*avOT*			
Middle frontal gyrus	−44 26 12	0.68	Superior/middle temporal gyrus [−62 −52 12]; CL = 0.83
	−50 28 22	0.72	
IFG: pars orbitalis	−42 26 −14	0.67	
Dorsomedial thalamus	−2 −8 10	0.70	
